# Relationship Between High-Density Lipoprotein-Cholesterol and Red Cell Distribution Width in Patients With Coronary Artery Disease

**DOI:** 10.7759/cureus.23132

**Published:** 2022-03-13

**Authors:** Hassan Raza, Tayyab Noor, Shereen Umer, Mavra Fatima, Ayisha Imran, Nomaan Malik

**Affiliations:** 1 Hematology, Chughtai Lab Lahore, Lahore, PAK

**Keywords:** atherosclerosis, immune inflammatory disease, red cell distribution width, high density lipo-protein cholesterol, coronary artery disease

## Abstract

Background

A high red cell distribution width (RDW), which indicates ongoing inflammation, and low levels of high-density lipoprotein-cholesterol (HDL-C) are associated with increased mortality and morbidity in patients with coronary artery disease (CAD). Recent studies have suggested that HDL-C possesses anti-inflammatory and antioxidant effects, which may explain its anti-atherogenic properties. This study aims to determine the relationship between HDL-C levels and RDW in patients with CAD.

Materials and methods

This cross-sectional study was performed on 120 patients with CAD from July 2020 to June 2021 in the Hematology Department of Chughtai Lab Lahore. Patients were graded according to the degree of coronary artery stenosis as follows: Grade 1,30%-50%; Grade 2, 51%-70%; and Grade 3,>70%. The HDL-C level was measured from venous blood samples by a fully automated Abbot Alinity analyzer. The RDW was measured by Sysmex XN-5000. The sample size was calculated using the Select Statistics calculator. The mean RDW and HDL-C of the patients were calculated, and correlation analyses were performed using the Pearson correlation coefficient.

Results

The HDL-C level was inversely related to the RDW. Of the 120 patients, 38, 44, and 38 had Grade 1, Grade 2, and Grade 3 stenosis, respectively. The mean HDL-C level and RDW were 30.58 ±3.77 mg/dL and 16.04% ±1.66%, respectively. The value of r was −0.8622 (strongly negative). Data were stratified based on the degree of stenosis. The values of r in Grades 1, 2, and 3 were −0.43 (moderately negative), −0.604 (moderately negative), and −0.27 (weakly negative), respectively.

Conclusion

The RDW can be used as an additional marker to determine the disease status in CAD patients.

## Introduction

Coronary artery disease (CAD), which is commonly caused by atherosclerosis (AS), is the leading cause of death worldwide. Meanwhile, AS is a systemic lipid-driven immune inflammatory disease. Inflammation, which may be local or systemic, is one of the causes of CAD. The effect of leukocytes on the stability of atherosclerotic plaques makes them crucial in the pathophysiology of CAD. Initially, leukocytes permeate the endothelium and become activated when they reach the tunica intima. They cause microvascularity to develop there, which makes plaques more likely to rupture [1].

The red cell distribution width (RDW), which is determined from a conventional complete blood count (CBC), is a measure of the variability in the size of circulating erythrocytes and is expressed as the erythrocyte size coefficient of variation [2]. The RDW is calculated by dividing the standard deviation by the mean corpuscular volume (MCV) x100; the normal value is 11%-15%. A patient whose RDW is greater than 15% is diagnosed as having anisocytosis [3].

The RDW can be used as a biomarker in the diagnosis and prognosis of patients with CAD. However, the mechanisms underlying the relationship between RDW and CAD prognosis remain unknown. Recently, a high RDW has been linked to the course and severity of cardiovascular disorders [4,5]. Several pathogenetic pathways, including microvascular dysfunction, anemia, oxidative stress, high cholesterol, nutritional deficiency, renal dysfunction, and hyper-adrenergic tone, have been hypothesized as responsible for this mechanism [6-9]. In addition, a high RDW was also associated with increased risk for hypertension, atrial fibrillation, myocardial infarction, heart failure, stroke, and mortality [10].

Lifestyle changes, urbanization, and an accelerated aging process have caused a CAD epidemic in Pakistan. Hence, the incidence of CAD will continue to rise in the following decades. Currently, cardiovascular illnesses account for at least half of all fatalities in cities [11]. The use of percutaneous coronary stent implantation in patients with CAD is becoming more common as medical technology develops. As a result, discovering new prognostic markers for CAD has gained interest [12].

It is well understood that inflammation plays a significant role in AS. A number of studies have established that humoral indicators of inflammation are linked with the origin, development, and instability of atherosclerotic plaques as well as with future cardiovascular events [13].

It is well known that a low high-density lipoprotein-cholesterol (HDL-C) level is a strong risk factor for the development of CAD. The anti-atherogenic effects of HDL-C are attributed to its antioxidant and anti-inflammatory properties. Hence, it can be used as an independent predictor of CAD due to the atherosclerotic process. However, few studies have reported the relationship between a low HDL-C level and a high RDW. Therefore, the objective of this study is to determine the correlation of the HDL-C level with RDW. Due to the anti-inflammatory properties of HDL-C, it can be used together with RDW to predict inflammation. In our hypothesis, we proposed an inverse relationship between serum HDL-C levels and RDW.

## Materials and methods

This study included 120 patients who were newly diagnosed with CAD in Chughtai Lab Lahore from July 2020 to June 2021. The patients were graded according to the degree of stenosis of the coronary artery. Patients with stenosis ranging from 30% to 50% were classified as having Grade 1 stenosis, from 51% to 70% as Grade 2 stenosis, and greater than 70% as Grade 3 stenosis. Patients with a normal coronary angiogram, coronary artery stenosis less than 30%, histories of an acute coronary syndrome (ACS), cardiac surgery, severe valve disease, kidney failure, significant anemia (hemoglobin <9 mg/dL), and blood transfusion within the previous three months, and conditions such as thalassemia, hemophilia, or taking lipid-lowering medications were excluded from the study.

The study was approved by the institutional review board of our institution (CIP-IRB-1049). Following informed consent, the medical history of the patients was obtained, and clinical examinations were conducted. Additionally, the HDL-C level and RDW were obtained. For HDL-C, a 3-mL venous blood sample was collected in a lithium heparin vial at least 10 hours after the patient had fasted. A fully automated Abbot Alinity analyzer was used to measure the HDL-C levels in venous blood samples. For RDW, a 1-mL blood sample was collected and dispensed in an EDTA vial. An automated hematological analyzer (Sysmex XN-5000 ) was used to calculate the RDW. 

The sample size was calculated by using the Select Statistics sample size calculator (http://select-statistics.co.uk/calculators/), where a confidence level of 95% and a margin of error of 5.5% were entered. The mean and standard deviation were calculated, and frequencies were indicated by percentages. Correlation analyses were performed using the Pearson Correlation coefficient. All analyses were performed with SPSS for version 23.0 (IBM Corp., Armonk, NY).

## Results

In this cross-sectional study, 120 newly diagnosed patients with CAD were enrolled to determine the relationship between the HDL-C level and RDW. Patients were categorized according to their age; 22 (18.3%) were ≤50 years and 98 (81.6%) were >50 years. Sixty-four (53.3%) patients were males while 56 (46.6%) were females. Regarding the severity of CAD, 38 (31.6%), 44 (36.7%), and 38 (36.7%) had Grades 1, 2, and 3 stenoses, respectively, as shown in Table 1. 

**Table 1:** Details of all the variables CAD, coronary artery disease

Variable		No. of patients	Percentage
Age	≤ 50 yrs	22	18.3%
	> 50 yrs	98	81.6%
Gender	Male	64	53.3%
	Female	56	46.6%
Severity of CAD	Grade 1	38	31.6%
	Grade 2	44	36.7%
	Grade 3	38	31.7%

The mean HDL-C level and RDW among all patients were 30.58 ±3.77 mg/dL and 16.04% ± 1.66%, respectively. The Pearson correlation coefficient (r) was calculated to show the relationship between the HDL-C level and RDW; the value of r was −0.8622 as shown in Table 2. The results revealed a strong and negative correlation between the serum HDL level and RDW.

**Table 2:** Correlation between mean HDL-C level and RDW The r-value was determined by the Pearson correlation coefficient, with a p-value < 0.05 as significant. HDL-C, high-density lipoprotein cholesterol; RDW, red blood cell distribution width; SD, standard deviation

Variables	Mean ± SD	r value	P-value
HDL (mg/dL)	30.6 ± 3.77	−0.86	<0.05
RDW (CV) %	16.04 ± 1.66		

Data were stratified based on the degree of stenosis of the coronary artery. Patients of Grade 1 had a mean HDL-C level of 35.54 ±1.10 mg/dL with a mean RDW value of 13.33 ± 0.30%. The mean HDL-C levels in Grade 2 cases were 26.42 ± 1.62 mg/dL with a mean RDW value of 17.82 ± 0.49, whereas the mean HDL-C levels in Grade 3 cases were 29.84 ±0.83 mg/dL with an RDW value of 15.52 ± 0.42%. Overall, there was an inverse correlation between serum HDL and RDW in the different CAD severity groups. All the disease categories showed an inverse relationship between HDL-C levels and RDW with Grade 2 having the strongest inverse relationship (r = −0.60) followed by Grade 1 (r = −0.43) and 3 (r = −0.27) (Table 3).

**Table 3:** Stratification of data in terms of severity of CAD and correlation of HDL with RDW The r-value was determined by the Pearson correlation coefficient, with a p-value < 0.05 as significant. CAD, coronary artery disease; HDL, high-density lipoprotein, RDW, red cell distribution width; SD, standard deviation.

Severity of CAD		Variables	Mean ± SD	r value	P-value
Grade 1	HDL (mg/dL)		35.84± 1.10	−0.4367	<0.05
	RDW (%)		13.33± 0.30		
Grade 2	HDL (mg/dL)		26.42± 1.62	−0.6043	<0.05
	RDW (%)		17.82± 0.49		
Grade 3	HDL (mg/dL)		29.95±0.83	−0.2751	<0.05
	RDW (%)		15.52± 0.42		

## Discussion

The RDW is one of the parameters used to diagnose and classify various types of anemias. Many studies have suggested that the RDW can also be used as a prognostic factor in various types of disorders [14]. These include cardiovascular diseases such as AS, myocardial infarction, and even heart failure. Other markers with considerable value in determining prognosis include mean platelet volume, total leukocyte count, monocyte-to-HDL ratio, and the platelet-to-lymphocyte ratio [15]. There is evidence that the RDW is linked to the development of cardiovascular disease [16].

According to Patel et al., RBCs with RDWs greater than 14% have substantially decreased ability to deform when going through small blood vessels, which can disturb blood flow and lead to a decrease in tissue oxygen delivery. This may explain the higher risk of unfavorable cardiac and vascular events linked with a high RDW [17]. According to Arbel et al., patients with a high RDW have an increased risk of cardiac morbidity and mortality, irrespective of their anemia status [18]. 

The association of a high RDW with peripheral vascular disorders, chronic respiratory disorders, chronic kidney diseases, thromboembolism, and even sepsis has been proven by many studies [19]. In a study by Lippi et al., they demonstrated that the combination of RDW with troponin T improved the diagnostic sensitivity of ACS to 99%, meaning that the combined parameters were more effective at detecting ACS than troponin T alone. In addition, they also concluded that the RDW of a patient with acute MI can be used to predict the severity of CAD [20].

Similarly, in another study, the RDW was shown to be inversely related to the hemoglobin level and MCV and directly related to age. After comparing lipid values across RDW categories adjusted for age, hemoglobin level, and MCV, the RDW was found to be negatively associated with HDL-C levels and positively associated with the atherogenic index of plasma, triglyceride levels, and total cholesterol-to-HDL ratio in women [21]. In another study that determined the prognostic value of the RDW, the authors reported an increased mortality rate among patients with above-normal RDWs [22].

As a prognostic indicator in ICU patients, the RDW aids in the decision-making process for early and effective therapeutic intervention as well as early discharge by stratifying patients upon admission. Furthermore, as the RDW is frequently assessed as part of the admitting CBC analysis, there are no additional costs. The RDW may be included in the severity sickness score in future investigations. Our results, as well as their limitations, present an essential focus for future translational research [23].

Many cross-sectional and retrospective studies have been conducted to investigate the relationship between a high RDW value and in-/out-of-hospital mortality in various groups. In addition to other inflammatory markers and scores, the RDW was found to be an affordable prognostic marker and predictor of significant morbidity, which can help in decision making and overall management of patients, particularly those in intensive care units [24].

Limitations

A few limitations exist in our study. For instance, this was a single-center study that included a limited population. Another limitation is that no follow-up data were available to assess the impact of the RDW on treatment or prognosis. Thus, it is necessary to conduct further studies to determine the prognostic role of the RDW in the management of patients with CAD.

## Conclusions

The present study revealed an inverse relationship between HDL-C levels and RDW in patients with CAD, providing further insight into the disease pathophysiology. As there is currently no available inflammatory marker for CAD, the RDW can be used as an additional marker to determine the disease severity and prognosis. This may help in the decision-making and management of patients. We recommended that further studies involving more inflammatory markers be conducted in multiple tertiary care settings to reach a valuable conclusion.

## References

[1] Budzianowski J, Pieszko K, Burchardt P, Rzeźniczak J, Hiczkiewicz J (2017). The role of hematological indices in patients with acute coronary syndrome. Dis Markers.

[2] Clarke K, Sagunarthy R, Kansal S (2008). RDW as an additional marker in inflammatory bowel disease/undifferentiated colitis. Dig Dis Sci.

[3] Danese E, Lippi G, Montagnana M (2015). Red blood cell distribution width and cardiovascular diseases. J Thorac Dis.

[4] Yousefi B, Sanaie S, Ghamari AA, Soleimanpour H, Karimian A, Mahmoodpoor A (2020). Red cell distribution width as a novel prognostic marker in multiple clinical studies. Indian J Crit Care Med.

[5] Li N, Zhou H, Tang Q (2017). Red blood cell distribution width: a novel predictive indicator for cardiovascular and cerebrovascular diseases. Dis Markers.

[6] Reinhart WH, Piety NZ, Goede JS, Shevkoplyas SS (2015). Effect of osmolality on erythrocyte rheology and perfusion of an artificial microvascular network. Microvasc Res.

[7] Xu D, Murakoshi N, Sairenchi T (2015). Anemia and reduced kidney function as risk factors for new onset of atrial fibrillation (from the Ibaraki prefectural health study). Am J Cardiol.

[8] Tziakas D, Chalikias G, Grapsa A, Gioka T, Tentes I, Konstantinides S (2012). Red blood cell distribution width: a strong prognostic marker in cardiovascular disease: is associated with cholesterol content of erythrocyte membrane. Clin Hemorheol Microcirc.

[9] Yoon HE, Kim SJ, Hwang HS (2015). Progressive rise in red blood cell distribution width predicts mortality and cardiovascular events in end-stage renal disease patients. PLoS One.

[10] Buyukkaya E, Erayman A, Karakas E (2016). Relation of red cell distribution width with dipper and non-dipper hypertension. Med Glas (Zenica).

[11] Chen WW, Gao RL, Liu LS (2017). China cardiovascular diseases report 2015: a summary. J Geriatr Cardiol.

[12] Li H, Sun K, Zhao R (2017). Inflammatory biomarkers of coronary heart disease. Front Biosci (Landmark Ed).

[13] Hu L, Li M, Ding Y (2017). Prognostic value of RDW in cancers: a systematic review and meta-analysis. Oncotarget.

[14] Li H, Sun K, Zhao R (2018). Inflammatory biomarkers of coronary heart disease. Front Biosci (Schol Ed).

[15] Poz D, De Falco E, Pisano C, Madonna R, Ferdinandy P, Balistreri CR (2019). Diagnostic and prognostic relevance of red blood cell distribution width for vascular aging and cardiovascular diseases. Rejuvenation Res.

[16] Haybar H, Pezeshki SM, Saki N (2019). Evaluation of complete blood count parameters in cardiovascular diseases: an early indicator of prognosis?. Exp Mol Pathol.

[17] Patel KV, Mohanty JG, Kanapuru B, Hesdorffer C, Ershler WB, Rifkind JM (2013). Association of the red cell distribution width with red blood cell deformability. Adv Exp Med Biol.

[18] Arbel Y, Raz R, Weitzman D (2013). Red blood cell distribution width and the risk of cardiovascular morbidity and all-cause mortality: a population-based study. Eur Heart.

[19] Söderholm M, Borné Y, Hedblad B, Persson M, Engström G (2015). Red cell distribution width in relation to incidence of stroke and carotid atherosclerosis: a population-based cohort study. PLoS One.

[20] Lippi G, Filippozzi L, Montagnana M, Salvagno GL, Franchini M, Guidi GC, Targher G (2009). Clinical usefulness of measuring red blood cell distribution width on admission in patients with acute coronary syndromes. Clin Chem Lab Med.

[21] Lippi G, Sanchis-Gomar F, Danese E, Montagnana M (2013). Association of red blood cell distribution width with plasma lipids in a general population of unselected outpatients. Kardiol Pol.

[22] Lazzeroni D, Moderato L, Marazzi PL (2021). Red blood cell distribution width as a novel prognostic marker after myocardial revascularization or cardiac valve surgery. Sci Rep.

[23] Chu Y, Yuan Z, Meng M, Zhou H, Wang C, Yang G, Ren H (2017). Red blood cell distribution width as a risk factor for inhospital mortality in obstetric patients admitted to an intensive care unit: a single centre retrospective cohort study. BMJ Open.

[24] Safdar SA, Modi T, Sriramulu LD (2017). The role of red cell distribution width as a predictor of mortality for critically ill patients in an inner-city hospital. J Nat Sci Biol Med.

